# Fabrication of Functionalized Double-Lamellar Multifunctional Envelope-Type Nanodevices Using a Microfluidic Chip with a Chaotic Mixer Array

**DOI:** 10.1371/journal.pone.0039057

**Published:** 2012-06-18

**Authors:** Katsuma Kitazoe, Yeon-Su Park, Noritada Kaji, Yukihiro Okamoto, Manabu Tokeshi, Kentaro Kogure, Hideyoshi Harashima, Yoshinobu Baba

**Affiliations:** 1 Department of Applied Chemistry, Graduate School of Engineering, Nagoya University, Nagoya, Japan; 2 FIRST Research Center for Innovative Nanobiodevices, Nagoya University, Nagoya, Japan; 3 Department of Biophysical Chemistry, Kyoto Pharmaceutical University, Kyoto, Japan; 4 Laboratory for Molecular Design of Pharmaceutics, Faculty of Pharmaceutical Sciences, Hokkaido University, Sapporo, Japan; 5 Health Technology Research Center, National Institute of Advanced Industrial Science and Technology, Takamatsu, Japan; Texas A&M University, United States of America

## Abstract

Multifunctional envelope-type nanodevices (MENDs) are very promising non-viral gene delivery vectors because they are biocompatible and enable programmed packaging of various functional elements into an individual nanostructured liposome. Conventionally MENDs have been fabricated by complicated, labor-intensive, time-consuming bulk batch methods. To avoid these problems in MEND fabrication, we adopted a microfluidic chip with a chaotic mixer array on the floor of its reaction channel. The array was composed of 69 cycles of the staggered chaotic mixer with bas-relief structures. Although the reaction channel had very large Péclet numbers (>10^5^) favorable for laminar flows, its chaotic mixer array led to very small mixing lengths (<1.5 cm) and that allowed homogeneous mixing of MEND precursors in a short time. Using the microfluidic chip, we fabricated a double-lamellar MEND (D-MEND) composed of a condensed plasmid DNA core and a lipid bilayer membrane envelope as well as the D-MEND modified with trans-membrane peptide octaarginine. Our lab-on-a-chip approach was much simpler, faster, and more convenient for fabricating the MENDs, as compared with the conventional bulk batch approaches. Further, the physical properties of the on-chip-fabricated MENDs were comparable to or better than those of the bulk batch-fabricated MENDs. Our fabrication strategy using microfluidic chips with short mixing length reaction channels may provide practical ways for constructing more elegant liposome-based non-viral vectors that can effectively penetrate all membranes in cells and lead to high gene transfection efficiency.

## Introduction

Gene therapy is an administration of genes within cells to treat diseases by correcting defective genes that cause the diseases. It has attracted great attention among scientific research, engineering, and clinical application communities because of its huge potential for effective treatments of various diseases ranging from congenital genetic disorders to lifestyle diseases. In general, gene therapy requires gene carriers, called gene delivery vectors (hereafter referred to as ‘vectors’ for simplicity), to deliver genes within target cells. Mainly two different types of vectors exist: viral and non-viral vectors. Viral vectors, such as adenoviruses and retroviruses, offer high gene transfection efficiency but they come with a high health risk due to the inability to control their immunogenicity and pharmacokinetics [1]–[3]. Non-viral vectors, represented by various liposome and polymer vectors, can be designed with suppressed toxicity and antigenicity by controlling their physical properties but they have disadvantages of instability in the blood stream, meager specificity toward targets, and low gene transfection efficiency [1]–[4].

Recently non-viral vectors have gained much more attention than viral vectors, despite their many problems-to-be-solved, due to their relatively low health hazard, capability for drug delivery, which is infeasible with viral vectors, and high potential for order-made gene therapy [1]–[4]. Liposome-based non-vial vectors have been especially attractive because liposomes are composed of biological components which lead to their low toxicity and low antigenicity; they can be metabolized *in vivo*; and they can encapsulate enzymes and drugs inside themselves [3], [5]–[9]. Many investigations on liposome-based non-viral vectors have focused on fabrication of multifunctional liposomes such as temperature-sensitive liposomes [7], ganglioside- and sphingomyelin-modified liposomes with long-term retention capability in blood [5], pH-sensitive liposomes releasing encapsulated materials in endosomes in a low pH environment [6], magnetic liposomes [8], and ultrasound-sensitive liposomes [9].

For efficient gene delivery to cell nuclei, it is essential to construct non-viral vectors that can effectively break through all barriers in cells. For this, an individual non-viral vector should be systematically packed with desired functional elements so that all the elements work properly and effectively. Focusing on those matters, Kogure et al. [10] introduced a novel non-viral vector which consisted of a DNA-polycation condensate core and a lipid bilayer membrane envelope surrounding the core. This kind of non-viral vector, called a multifunctional envelope-type nanodevice (MEND), allows programmed packaging of various functional elements into an individual nanostructured liposome. The core of such a double-lamellar MEND (D-MEND) can accommodate various gene therapy molecules, such as DNAs [10]–[15], siRNAs [16]–[19] or proteins [20], as condensates with polycations. The lipid bilayer membrane envelope of the MEND provides both structural stability and topological control, and can be further modified with functional elements, such as polyethylene glycols [18], [21], octaarginine [12]–[17], and transferrin [11], to enhance intercellular trafficking. Akita et al. [22] have introduced a tetra-lamellar MEND (T-MEND) that focused on efficient nuclear membrane penetration. In their T-MEND, a DNA-polycation condensate core was coated with two nuclear membrane-fusogenic inner lipid envelopes and two endosome-fusogenic outer lipid envelopes. Stepwise fusion of the two different lipid membranes allowed highly efficient transgene expression in non-dividing cells. Gene delivery by such MENDs has proven to be highly stable and efficient [19], [21], [22], brightening up their future as non-viral vectors.

Conventionally MENDs have been fabricated by wet chemistry-based bulk batch methods such as lipid film hydration and detergent-rich small unilamellar vesicle (SUV)-fusion [10]–[22], with the latter method being more successful recently [11], [22]. In the vesicle formation process from micelles, detergent-rich SUVs are temporarily formed by detergent removal and they can fuse with each other to form large unilamellar vesicles by complete removal of detergent [23]. The conventional fabrication methods involve largely two different types of processes: the polycation-induced condensation of nucleic acids (or proteins) and the formation of one or more lipid bilayer membrane envelopes on each condensate core. However, the conventional methods are complicated, labor-intensive, and time-consuming, require relatively large quantities of raw materials, and produce relatively large amounts of waste. Furthermore, such bulk batch methods are unsuitable for fabricating small quantities of diverse MENDs with different functionalities in a short time, which is essential for their effective application to order-made gene therapy.

Fabrication of MENDs using microfluidic chips can be an excellent way for overcoming these problems because this lab-on-a-chip approach allows utilization of the intrinsic merits of microfluidic devices [24]–[29], such as their short reaction time, high reaction efficiency, and low raw material consumption. Previously we reported a seminal work on the application of microfluidic chips to the fabrication of DNA-polycation condensate cores and MENDs [30]. Plasmid DNA-ploy-L-lysine condensate cores and MENDs were fabricated in a simple microfluidic chip composed of three straight inlet channels and one straight smooth reaction channel allowing diffusion-based precursor mixing.

Unfortunately, fabrication of MENDs in the microfluidic chip was unreliable. Particle size of the MENDs decreased as their precursor flow rate increased and reproducibility of their fabrication (i.e., their size and the size distribution) was poor. These could be mainly attributed to the diffusion-based poor and inconsistent mixing of their precursors in the smooth reaction channel. Thus, for reliable on-chip fabrication of MENDs the most reasonable but important approach is to use microfluidic chips with novel designs that allow homogeneous precursor mixing in a short time. A microfluidic chip with an array of bas-relief structures on the floor of its reaction channel may be suitable for this purpose [31]. Such a chaotic mixer array generates chaotic flows in the reaction channel and that can lead to homogeneous mixing of the precursor solutions in a short time.

**Figure 1:**
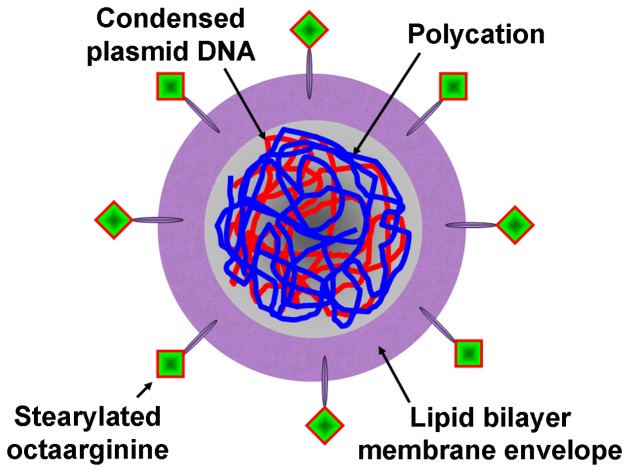
Schematic representation of a D-MEND. It consists of a condensed DNA/polycation core and a lipid bilayer membrane envelope. Its surface is further modified with the membrane penetrating peptide stearylated octaarginine to improve cellular uptake and intracellular trafficking.

Here, we report the reliable fabrication of D-MENDs using a microfluidic chip that had an array of a staggered chaotic mixer with bas-relief structures on the floor of its reaction channel. The chaotic mixer array allowed homogeneous precursor mixing and that led to reliable results in fabricating D-MENDs as well as D-MENDs functionalized further with trans-membrane peptides (Figure 1). Effects of the precursor flow rate on the size and zeta potential of those non-viral vectors were also investigated. In addition, the results from the on-chip fabrications were compared with corresponding results from bulk batch fabrications.

## Results and Discussion

### Microfluidic Chips

**Figure 2:**
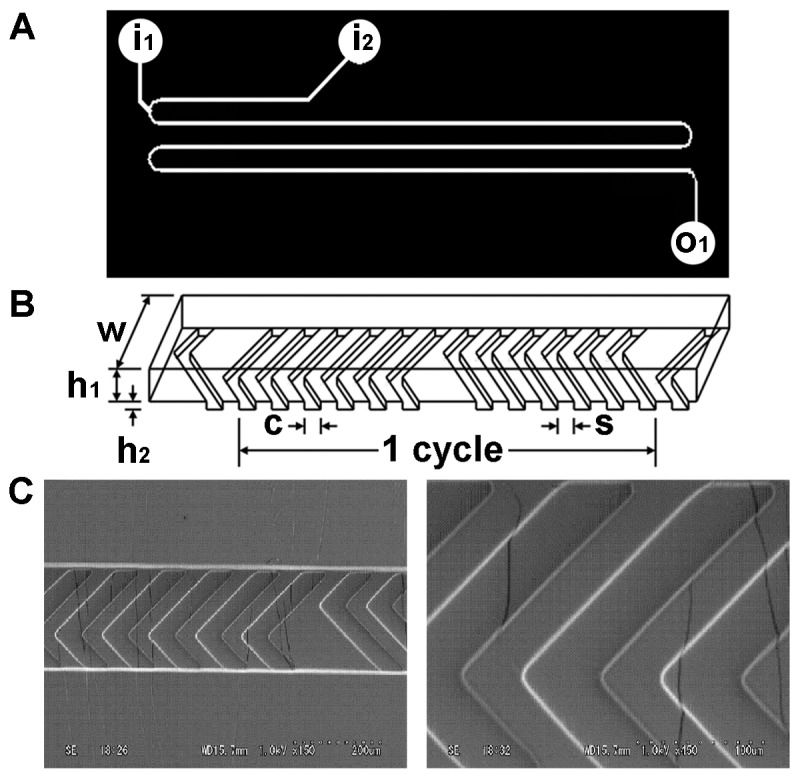
Structure of the microfluidic chip with a chaotic mixer array. (A) Two-dimensional design of the chip: i_1_, an inlet for f-NA and STR-R8; i_2_, an inlet for SUV and D-MEND; o_1_, an outlet. (B) Schematic diagram of the staggered chaotic mixer: h_1_=75 µm, w=200 µm, h_2_=25 µm, c=50 µm, s=50 µm. (C) SEM images of the parts of the staggered chaotic mixers and herringbone ridges in the reaction channel: 150× (left) and 450× (right). The SEM images show the fabricated staggered chaotic mixer array and herringbone ridges.

Figure 2 shows schematic diagrams of a microfluidic chip and its reaction channel with a chaotic mixer array. The chip, made of polydimethylsiloxane (PMDS), was fabricated by a soft lithography technique [32]. The design of the chaotic mixer array on the floor of the reaction channel was slightly modified from that reported by Stroock et al. [31]. As shown in Figure 2A, the chip consisted of a long reaction channel (three lanes), two inlet channels, and one outlet channel. The two inlet channels were connected to one end of the reaction channel; the outlet channel was connected to the other end of the reaction channel. The reaction channel had an array of 69 cycles of the staggered chaotic mixer with two sequential sets of herringbone structures. Each herringbone set had six ridges and the direction of their asymmetry switched from one set to the other. Details on the structural dimensions of the chaotic mixer array are shown in Figure 2B: channel height=75 µm; channel width=200 µm; ridge height=25 µm; ridge width=50 µm; space between adjacent two ridges=50 µm. The ridge height was fairly larger than that of ∼17.7 µm for the reference designs [31] and that might ensure better degree of precursor mixing. The scanning electron microscopy (SEM) images in Figure 2C confirm the staggered chaotic mixer array and herringbone ridges were fabricated on the floor of the reaction channel.

### SUVs and Functional Nucleic Acid Cores

**Figure 3:**
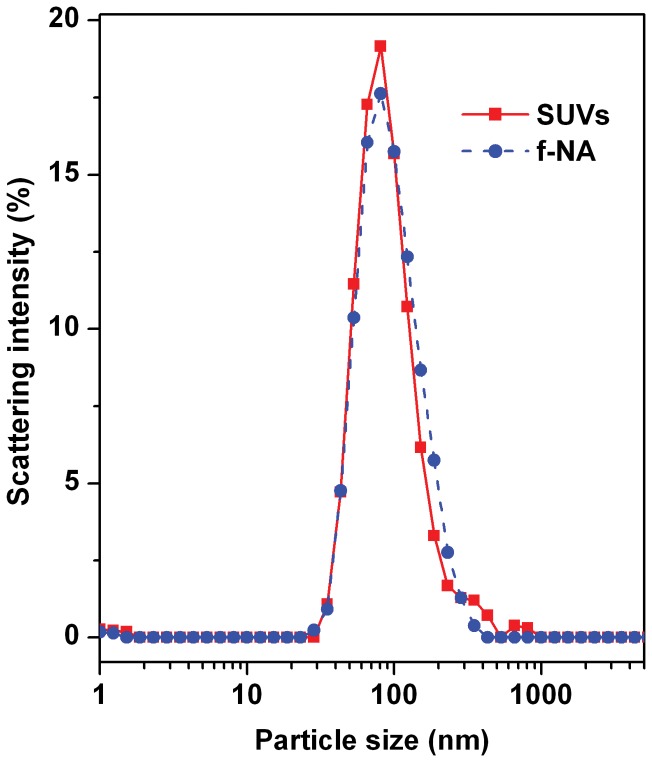
DLS particle size distribution profiles for SUVs and f-NAs. The sharp main peak for the SUVs (red) indicates they were formed with a narrow particle size distribution (*d*_average_=104.8 nm, *d*_predominant_=81.1 nm). The sharp main peak for f-NAs (blue) suggests they also had a narrow particle size distribution (*d*_average_=102.3 nm, *d*_predominant_=81.1 nm).

SUVs were prepared by drying a phospholipid solution of 1,2-dioleoyl-*sn*-glycero-3-phosphoethanolamine (DOPE), cardiolipin (CL), and chloroform, subsequently hydrating it using 4-(2-hydroxyethyl)-1-piperazineethanesulfonic acid (HEPES) buffer, then incubating the hydrated solution, and finally ultrasonically treating it. The SUVs prepared in the bulk solution were analyzed using dynamic light scattering (DLS). Figure 3 shows their particle size distribution profile (red line). The fairly sharp large main peak indicated the formation of SUVs with a fairly narrow particle size distribution. Their average particle size and predominant particle size (the particle size with strongest scattering intensity) were estimated to be 104.8 nm and 81.1 nm, respectively: this size difference was due to the presence of very large particles (i.e., 200–1000 nm) formed together during the preparation. Their zeta potential was measured as −44.6 mV. This highly negative *ζ* value implied that the SUVs could favorably form complexes with positively charged functional nucleic acid (f-NA) cores in subsequent MEND fabrication. The size, size distribution, and *ζ* value suggested that those SUVs might be suitable for use as a MEND precursor.

f-NA cores were prepared by mixing a plasmid DNA (pcDNA-DEST53 Gateway vector) solution and a protamine (protamine sulfate, salmon milt) solution followed by forming the vortex and incubating it at room temperature. Figure 3 also shows their particle size distribution profile (blue line). The fairly sharp large main peak suggested they were relatively small sized and with a fairly narrow size distribution. Their average and predominant particle sizes were estimated to be 102.3 nm and 81.1 nm, respectively; this size difference could be attributed to the contribution from the larger particles (i.e., 200–500 nm) formed during the preparation. These sizes were within a typical size range (72.3−184 nm [15]) for DNA-polycation condensates used for fabricating various MENDs that have been successfully applied to gene transfection. Their zeta potential was measured as +31.5 mV. This positive *ζ* value implied that the negatively charged plasma DNAs and the positively charged protamins interacted electrostatically to form the f-NAs. The DNAs with relatively mild negative charge were easily condensed by the protamines with strong positive charge (arginine content >70%) and that resulted in the formation of f-NA cores with the net positive *ζ* value. The size, size distribution, and *ζ* value suggested that those f-NAs could be successfully used as a MEND precursor.

### Mixing in the Chaotic Mixer Array

**Figure 4:**
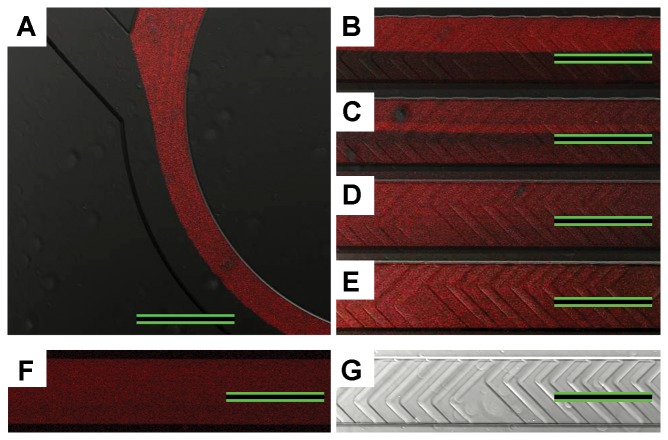
Fluorescence images tracing the progress of the MEND precursor mixing in the microchannel. The rhodamine-stained SUV solution and the f-NA solution were mixed in the microchannel and fluorescence images (*xy*-plane) were recorded using a confocal microscope in (A) 0, (B) 1, (C) 5, (D) 10, and (E) 15 cycle regions of the chaotic mixer array. (F) Accumulated scanned fluorescence images along the *z*-axis direction in the 15 cycle region. (G) Image (*xy*-plane) of the 15 cycle region before precursor introduction. Flow rate=10.0 µl min^−1^. Scale bars=300 µm. The fluorescence images indicate homogeneous mixing of the two precursor solutions in the early cycle region of the chaotic mixer array.

Confocal microscopy was employed to trace mixing of two precursor solutions in the reaction channel. The rhodamine-stained SUV solution and the f-NA core solution were introduced simultaneously into separate inlets of the microfluidic chip using microsyringe pumps and then fluorescence signals were observed in various regions of the reaction channel. Figure 4 shows fluorescence images for the precursor mixture solution flowing at the rate of 10.0 µL min^−1^ in various regions of the staggered chaotic mixer array. In the 0 cycle region (the smooth junction region between the two inlet channels without bas-relief structures), we observed no solution mixing due to laminar flow of the solutions. Fairly homogeneous fluorescence was observed in the entire 10 cycle region. In the entire 15 cycle region, practically homogeneous fluorescence was observed. Similar results were obtained when using slower solution flow rates of 1.0 and 5.0 µL min^−1^ (data not shown). Higher flow rates were avoided because they often led to poor reproducibility in the sizes and *ζ* values of resulting particles. These results indicated homogeneous mixing of the two precursor solutions in the early cycle region of the chaotic mixer array and that confirmed high efficiency of the chaotic mix array for the mixing of our MEND precursors. Homogeneous mixing of the precursor solutions was further confirmed by scanning along the *z*-direction in the 15 cycle region. The accumulated scanned fluorescence image in Figure 4F showed substantially uniform fluorescence along the *z*-axis of the reaction channel. All the above results suggested that the microfluidic chip enabled efficient homogeneous mixing of the precursor solutions and thus it could be used for MEND fabrication.

The efficient homogeneous mixing in our microfluidic chip could be mainly attributed to the chaotic mixer array on the floor of the reaction channel. In general, mixing efficiency of microfluidic chips can be characterized by the Péclet number (*Pe*) which is a measure of the relative rate of convective transport to diffusive transport in fluid flows: a smaller *Pe* is more favorable to mixing [31], [33]. The Péclet number is given as follows: *Pe*=*U*×*l/D* where *U* is average flow speed, *l* is cross-sectional dimension of the reaction channel, and *D* is a molecular diffusion coefficient [31]. For the precursors (*D* ∼1 µm^2^ s^−1^ for liposomes with *d* ∼100−200 nm [34−36]) flowing at 10 µL min^−1^ in our microfluidic chip (*l*=122.5 µm), *Pe* is ∼ 1.36 × 10^6^. This very large *Pe* indicates predominance of uniaxial flow which is unfavorable for mixing. In a simple smooth microchannel (height=75 µm, width=200 µm) for which the cross-sectional dimension is the same as that for our reaction channel, the distance along the channel required for mixing to occur (the mixing length, Δ*y* ∼ *Pe*×*l* [31]) is then ∼16700 cm. This very long mixing length implies that homogeneous mixing in the smooth microchannel requires very long reaction channel lengths. It will be extremely difficult to achieve homogeneous mixing in such a smooth reaction channel. However, in a microchannel with steady chaotic flow and high *Pe*, like in our reaction channel, the mixing length is defined as follows: Δ*y* ∼ *λ*×ln*Pe* where *λ* is a characteristic length determined by the geometry of trajectories in the chaotic flow [31]. Previously *λ* of ∼0.1 cm (0.08−0.13 cm, 90% mixing [31]) was reported for the microchannel with a chaotic mixer array with a structure that was very similar to our reaction channel structure. Thus, in our reaction channel with the chaotic mixer array, the mixing length for 90% mixing (Δ*y*_90_) at the flow rate of 10.0 µL min^−1^ was ∼ 1.41 cm. This very small Δ*y*_90_ suggested that a short length of the reaction channel might be enough to achieve homogeneous mixing.

Table 1 summarizes Péclet numbers and mixing lengths at the flow rates used in the experiments. In the flow rate range investigated, the Péclet numbers are very large. In such uniaxial flow conditions, extremely long lengths of smooth microchannels are required for achieving homogeneous mixing. In the smooth microchannel, the mixing length at the flow rate of 1 µL min^−1^ is 1670 cm and it increases linearly and sharply with increasing the flow rate. This observation indicates that increasing the flow rate in the smooth microchannel makes the mixing much more difficult. However, in our reaction channel with the chaotic mixer array, the mixing lengths were very small (1.18−1.41 cm), although the length increased slightly with increasing flow rate. The much reduced, small mixing lengths for our reaction channel, as compared with those for the smooth microchannel, suggested that the chaotic mixer array of the reaction channel was responsible for the observed efficient homogeneous mixing.

**Table 1:** The Péclet numbers and the mixing lengths in the microchannels (height=75 µm, width=200 µm) with and without the chaotic mixer array.

Flow rate (µL min^−1^)	1	3	5	7	10
Flow speed (cm s^−1^)	0.111	0.333	0.556	0.778	1.111
10^−5^×*Pe* a	1.36	4.08	6.81	9.53	13.6
Δ*y*, smooth (cm)^b^	1670	5000	8340	11700	16700
Δ*y*_90_, chaotic (cm)^c^	1.18	1.29	1.34	1.38	1.41

a Péclet number calculated taking *D*=1 µm^2^ s^−1^.

b Mixing length in the smooth microchannel.

c Mixing length in the microchannel with the chaotic mixer array.

### D-MENDs

D-MENDs were fabricated in the reaction channel of the microfluidic chip by introducing the f-NA core solution and the SUV solution into separate inlets and then they were analyzed using DLS. Various precursor flow rates were employed to investigate their effects on the size, size distribution, and *ζ* of resulting D-MENDs. In addition, D-MENDs were also fabricated by the bulk SUV-fusion method. The bulk batch-fabricated D-MENDs were analyzed using DLS for comparison with the results from the on-chip-fabricated D-MENDs.

**Figure 5:**
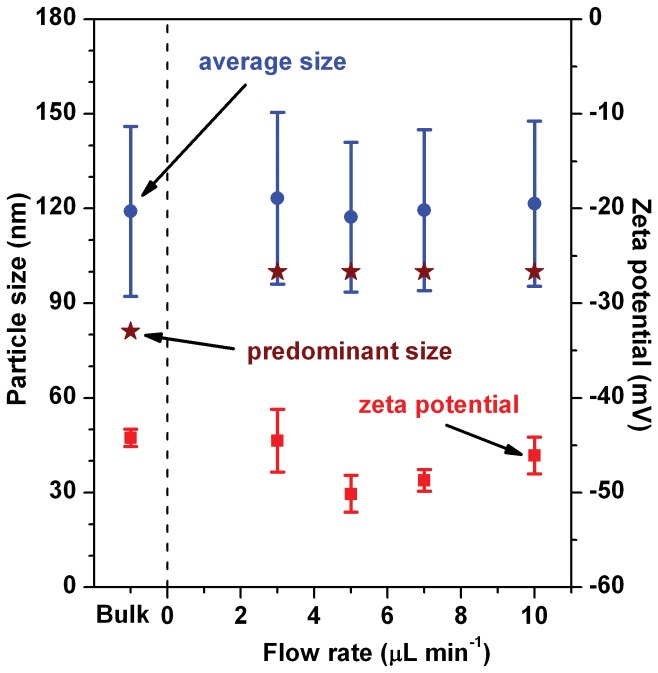
Precursor flow rate-dependent changes in the particle size and zeta potential of the D-MENDs. Both the average particle size (blue circles) and predominant particle size (wine red stars) of the D-MENDs depended only slightly on their precursor flow rate. Their zeta potential values (red squares) were highly negative due to their negatively charged lipid bilayer membrane envelope.

Figure 5 shows the average particle sizes (blue circles) and the predominant particle sizes (wine red stars) for the on-chip-fabricated D-MENDs at different precursor flow rates (see Figure S1, for corresponding DLS particle size distribution profiles) and Table 2 summarizes the results. The average particle sizes for the on-chip-fabricated D-MENDs were very similar to each other (117.3−123.2 nm) regardless of the flow rates. The average particle sizes for the D-MENDs were slightly larger than those for their precursors (SUVs=104.8 nm, f-NA core=102.3 nm) but very similar to the average size of 119.1 nm for the bulk batch-fabricated D-MENDs. In addition, the predominant particle size for the on-chip-fabricated D-MENDs was independent of their precursor flow rate. Their predominant particle size was 100.0 nm regardless of the flow rates, indicating that the majority of the on-chip-fabricated D-MENDs had a particle size of 100.0 nm. The differences between the average and predominant sizes could be ascribed to the partial destruction of D-MENDs followed by their combination. During on-chip fabrication, some of the initial formed D-MENDs could be partially destructed due to their collision onto the rough bas-relief structures on the floor of the long chaotic mixer array. One of them could combine with one or more of the intact D-MENDs or the partially destructed D-MENDs and that could result in larger-than-average-sized D-MENDs. The faster and stronger collisions (i.e., higher flow rates) might lead to more formation of much larger-than-average-sized D-MENDs. These observations on the particle sizes suggested relatively fast formation of D-MENDs with stable structures in the reaction channel. Previously use of a microfluidic chip with a simple smooth reaction channel (channel length=5.25 cm) led to the fabrication of D-MENDs for which particle size decreased with increasing their precursor flow rate from 1.0 to 10 µL min^−1^ [30]. This flow rate-dependent particle size change and the mixing lengths summarized in Table 1 suggest that the smooth reaction channel is unsuitable for the reliable fabrication of MENDs because a complete mixing of the precursors in such a channel requires very long channel lengths. Thus, the formation of similar-sized D-MENDs in our microfluidic chip at the very different precursor flow rates could be ascribed to the chaotic mixer array on the floor of the chip reaction channel. The chaotic mixer array led to very short mixing lengths (Table 1) and that ensured homogeneous mixing of the MEND precursor solutions in its early cycle region (i.e., in a short time) and hence the formation of the D-MENDs with stable structures.

**Table 2:** Average particle size, predominant particle size, and zeta potential of the D-MENDs and R8-MENDs.

Flow rate (µL min^−1^)	Bulk^a^	3	5	7	10
*d*_average, D-MEND_ (nm)	119.1	123.2	117.3	119.5	121.6
*d*_predominant, D-MEND_ (nm)	81.1	100.0	100.0	100.0	100.0
*ζ*_D-MEND_ (mV)	−44.2	−44.5	−50.1	−48.7	−46.1
*d*_average, R8-MEND_ (nm)	179.5	136.1	172.0	193.5	224.2
*d*_predominant, R8-MEND_ (nm)	152.0	100.0	100.0	100.0	100.0
*ζ*_R8-MEND_ (mV)	+45.0	+49.1	+50.1	+52.6	+47.1

a Data for the MENDs fabricated by the bulk SUV-fusion method.

Zeta potential, like the average particle size, for the on-chip-fabricated D-MENDs was little affected by their precursor flow rate, as shown in Figure 5 (red squares). Their *ζ* values (between −44.5 and −50.1 mV) were very similar to each other regardless of the flow rates (Table 2). The *ζ* values were very similar to the *ζ* value of −44.2 mV for the bulk batch-fabricated D-MENDs. These results indicated that the electrical charge structure on the surface of the on-chip fabricated D-MENDs was very similar to that of the bulk batch-fabricated D-MENDs.

The particle size and zeta potential observations we described above manifested the on-chip fabrication of the D-MEND composed of the f-NA core and the lipid bilayer membrane envelope. The highly negative *ζ* values which were similar to each other and the slightly increased particle sizes after the fabrication of the D-MENDs starting from f-NA cores implied that the positively charged f-NA core was encapsulated in the negatively charged lipid bilayer membrane envelope. Both the particle size and the electrical charge structure on the surface of the D-MENDs were slightly dependent on the fabrication methods and the precursor flow rates. The on-chip fabrication of the D-MENDs took about 10 min which was much faster than their bulk batch fabrication typically requiring about one day. The overall results here indicated that the microfluidic chip with the chaotic mixer array could be used for the fast, convenient, reliable fabrication of D-MENDs with a size and surface charge structure very similar to the size and charge structure of the bulk batch-fabricated D-MENDs.

### R8-MENDs

R8-MENDs were fabricated in the microfluidic chip to investigate the possibility for fast, convenient incorporation of additional functionalities onto D-MENDs. Stearylated octaarginine (STR-R8, a trans-membrane peptide) and the bulk batch-fabricated D-MENDs were concurrently introduced into the reaction channel through the respective inlets and that led to the addition of STR-R8 moieties on the phospholipid bilayer of the D-MENDs. Like the D-MEND fabrication, the on-chip fabrication of the R8-MENDs took about 10 min whereas their bulk batch fabrication took about one day.

**Figure 6:**
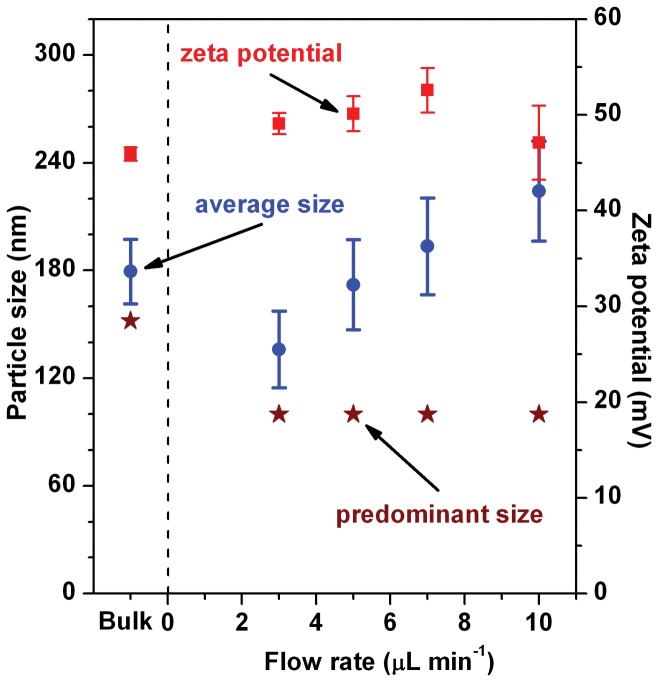
Precursor flow rate-dependent changes in the particle size and zeta potential of the R8-MENDs. As the precursor flow rate increased, their average particle size (blue circles) increased whereas their predominant particle size (wine red starts did not change. Their zeta potential values (red squares) were highly positive due to the presence of the positively charged STR-R8 on their surface.

Figure 6 shows the dependences of the average particle size (blue circles) and the predominant particle size (wine red stars) for the R8-MENDs on their precursor flow rate in the reaction channel (see Figure S2 in SI for corresponding DLS particle size distribution profiles) and Table 2 summarizes the results. The average particle size of the R8-MENDs fabricated at the flow rate of 3 µL min^−1^ was slightly larger than the 119.1 nm size for their precursor D-MENDs. However, the average particle size of the on-chip-fabricated R8-MENDs became larger as the flow rate increased. Interestingly their predominant particle size was independent of the precursor flow rate and was 100.0 nm regardless of the rate. The predominant particle size was slightly larger than of the 81.1 nm size for their precursor D-MENDs. In our bulk and on-chip fabrication conditions, the predominant particle sizes of the D- and R8-MENDs were always smaller than the average particle sizes of the corresponding MENDs.

Figure 6 also shows the dependence of zeta potential (red squares) for the R8-MENDs on their precursor flow rate. Zeta potential values for the on-chip-fabricated R8-MENDs were highly positive (between +47.1 and +52.6 mV, Table 2) and they were influenced only slightly by the precursor flow rates. The *ζ* values for the on-chip-fabricated R8-MENDs were very similar to the value for the bulk batch-fabricated R8-MENDs. These results indicated that the electrical charge structure on the surface of the on-chip-fabricated R8-MENDs was very similar to that of the bulk batch-fabricated R8-MENDs.

Our above observations on the particle sizes and zeta potential could be interpreted as follows. Both the average particle sizes and the predominant particle sizes for the on-chip-fabricated R8-MENDs were larger than corresponding sizes for their precursor D-MENDs, like the cases for the bulk batch-fabricated R8-MENDs. In addition, zeta potential changed from the highly negative values to highly positive values as the precursor D-MENDs were transformed to the R8-MENDs. These results implied that the mixing of the STR-R8 and D-MEND precursors in the reaction channel led to the successful formation of R8-MENDs having a surface covered by positively charged STR-R8. For the on-chip-fabricated R8-MENDs, the predominant particle size was 100.0 nm regardless of the precursor flow rate. This fact suggested that the majority of them had a particle size of 100.0 nm. The fact further indicated that the R8-MENDs with a particle size of 100.0 nm were the most stable R8-MEND species formed in our on-chip fabrication conditions. The observed flow rate-dependence of the predominant particle size contradicted to the flow rate-dependence of the average particle size. For on-chip-fabricated R8-MENDs, their average particle size became larger with increasing precursor flow rate. This trend for their average particle size could be attributed to the enhanced contribution from larger-than-average-sized R8-MENDs (see Figure S2 in SI for the corresponding DLS particle size distribution profiles). During on-chip fabrication of the R8-MENDs, their precursors would collide many times onto the rough bas-relief structures and the collisions became faster and stronger as the precursor flow rate increased. The collisions might naturally lead to partial destruction of the lipid bilayer membrane envelopes of the precursor D-MENDs and that resulted in the formation of partially destructed D-MENDs. In the reaction channel, larger sized R8-MENDs could be formed by the combination of two or more of the partially destructed D-MENDs with appropriate numbers of STR-R8. In addition, some of the initially formed R8-MENDs could collide onto the bas-relief structures and that might lead to the formation of partially destructed R8-MENDs. One or more of the partially destructed R8-MENDs could combine with one or more of the partially destructed D-MEND or R8-MENDs in the reaction channel and that subsequently led to larger-than-average-sized R8-MENDs. The faster and stronger collisions might lead to more formation of much larger-than-average-sized R8-MENDs. Thus, it might be highly desirable to use low precursor flow rates (i.e., ≤ 5 µL min^−1^) when fabricating R8-MENDs because low precursor flow rates were seen as highly beneficial to the fabrication of R8-MENDs with a narrow particle size distribution. Interestingly, the predominant particle size for the on-chip-fabricated R8-MENDs was much smaller than that for the bulk batch-fabricated R8-MENDs and their smaller particle size could be highly beneficial to their cell membrane penetration.

The overall results here indicated that the microfluidic chip with the chaotic mixer array in its reaction channel allowed for the convenient, fast, reliable fabrication of R8-MENDs having a size and surface charge structure comparable to or better than those of the R8-MENDs fabricated by the time-consuming, inconvenient bulk SUV-fusion method.

In conclusion, we successfully fabricated MENDs using a PDMS microfluidic chip with a chaotic mixer array in its reaction channel and then investigated their physical properties. The presence of the chaotic mixer array on the floor of the reaction channel dramatically reduced the mixing length in spite of very large *Pe* (>10^5^ at 1−10 µL min^−1^) favoring laminar flow. Their mixing lengths (<1.5 cm) were more than 1000 times smaller than those for the corresponding smooth reaction channel. The chaotic mixer array allowed homogeneous mixing of the precursor solutions in a short time (10–20 min) and that led to fast, convenient, reliable fabrication of D-MENDs as well as further functionalized R8-MENDs. Mixing of the phospholipid SUVs and the f-NAs in the microfluidic chip resulted in D-MENDs composed of a condensed plasmid DNA core and a lipid bilayer membrane envelope encapsulating the core. Their surface was strongly negatively charged due to the lipids. Similarly, mixing of trans-membrane peptide STR-R8 molecules and the D-MENDs in the microfluidic chip led to R8-MENDs in which STR-R8 moieties were added on the phospholipid bilayer of the D-MENDs. The MENDs were strongly positively charged due to the R8 moieties on their surface. The sizes and surface charge structures of the on-chip-fabricated MENDs were comparable to or better than those of the bulk batch-fabricated counterparts. Biological activities of those MENDs will be characterized in subsequent studies. Our results should greatly contribute to the development of fast, simple, convenient, economical, and reliable methods for fabricating various kinds of elegant liposome-based non-viral vectors that are suitable for practical gene therapy applications.

## Materials and Methods

### Materials

Acetone, isopropanol, chloroform, and ethanol were purchased from Wako Pure Chemical Industries (Japan). CL and trichloro(1*H*,1*H*,2*H*,2*H*-perfluorooctyl)silane were acquired from Sigma-Aldrich (USA). Silpot 184 and its catalyst were obtained from Dow Corning Toray (Japan). Protamine sulfate (from salmon milt) was obtained from Calbiochem (USA). HEPES buffer solution (10 mM, pH 7.4) was purchase from Dojindo (Japan). pcDNA-DEST53 Gateway vector was acquired from Invitrogen (USA). STR-R8 was obtained from Kurabo Industries (Japan). DOPE and 1,2-dipalmitoyl-*sn*-glycero-3-phosphoethanolamine-N-(lissamine rhodamine B sulfonyl) (ammonium salt) (18∶1 Liss Rhod PE, Rho-DOPE) were acquired from Avanti Polar Lipids (USA). SU-8 3050 and SU-8 developer were purchased from Microchem (Japan).

### Microfluidic Chip Fabrication

Microfluidic chips made of PMDS were fabricated using a soft lithography technique [32]. The design of their chaotic mixer array was adopted from that reported by Stroock et al. [31] after slight modifications. The most distinctive difference in the designs was in the height of herringbone ridges. We adopted much deeper ridges (25 µm vs. ∼17.7 µm for the reference designs [31]) to ensure better degree of precursor mixing.

A microfluidic chip master was fabricated as follows. First, a mirror-finished, 3-inch Si-wafer (Cretec Industry, Japan) was washed with acetone and isopropanol. The washed Si-wafer was placed on a hot plate and heated at 200°C for 5 min. After that, the wafer was placed on the rotor of a spin-coater (IH-D7, Mikasa, Japan) and then spin-coated (conditions: Slope 5 s, 500 rpm 5 s; Slope 8 s, 3000 rpm 45 s) with a photo-resist chemical SU-8 3050. After completing the spin-coating, the wafer was placed on the hotplate and heated at 95°C for 10 min. After cooling down to room temperature, the photo-resist chemical-coated wafer was masked with a transparency film printed with a microfluidic channel shape (Nagoya University Coop, Japan). After that, the photo-resist chemical was cured by exposing it to ultraviolet light for 15 s using photolithography equipment (MJB3, Karl Suss, Germany). After curing, the wafer was placed on the hotplate and heated at 95°C for 8 min. After that, the coated chemical was developed in Su-8 developer solution. Finally, the fabricated chip-master was washed thoroughly with isopropanol and its surface was silanized using trichloro(1*H*,1*H*,2*H*,2*H*-perfluorooctyl)silane.

A PDMS microfluidic chip was fabricated as follows. The silanized chip-master was placed in a Petri dish on a hot plate. PDMS precursor (Silpot 184, Dow Corning Toray, Japan) and its cross-linker were weighed (10∶1 weight ratio) separately and then mixed in a beaker. The mixture was then poured onto the Petri dish. After that, the dish was heated at 90°C for 2 h. After cooling down to room temperature, the fabricated PDMS structure was removed from the chip-master and then trimmed into a rectangular shape (∼76 mm×∼52 mm). The trimmed PDMS structure was punched to make inlet and outlet holes. Finally, the PDMS structure was attached on a slide glass (76 mm×52 mm, Matsunami Glass, Japan) to construct a microfluidic chip.

### f-NA and SUV Fabrication

A f-NA core solution was prepared by mixing plasmid DNA solution (0.10 mg mL^−1^) and 75 µL of 10 mM HEPES buffer solution containing protamine sulfate (0.10 mg mL^−1^) followed by forming the vortex for 30 s and incubating it at room temperature for 15 min. Electrostatic interaction between the negatively charged plasmid DNAs and the polycationic nuclear proteins led to the formation of their compact agglomerates called f-NAs.

SUVs were prepared as follows. First, a phospholipid solution was prepared by mixing 20 µL of DOPE in chloroform (20 mg mL^−1^), 165 µL of CL in ethanol (5 mg mL^−1^), and 165 µL of chloroform. After that, the solution was transferred to a brown test tube and dried by blowing nitrogen gas. After drying, 165 µL of chloroform was dropped into the test tube, dried by blowing nitrogen gas over the liquid surface, and then further dried under vacuum for 30 min in the dark. After drying was completed, 1 mL of 10 mM HEPES buffer (pH 7.4) was added and then the solution was incubated for 10 min at room temperature for sufficient hydration. After completing hydration, the solution was ultrasonically treated for 5 min, using a vortex-type sonicator (UH-50, SMT Inc., Japan), to complete the SUV preparation. Rhodamine-stained SUVs were prepared by following the same procedures for preparing SUVs described above except for using a phospholipid solution containing 20 µL of DOPE in chloroform (20 mg mL^−1^), 165 µL of CL in ethanol (5 mg mL^−1^), 41 µL of Rho-DOPE in chloroform (0.01 mg mL^−1^), and 165 µL of chloroform.

### Bulk Fabrication of MENDs

D-MENDs were fabricated by mixing 250 µL of the SUV solution and 125 µL of the f-NA core solution followed by forming the vortex and incubating it for 15 min at room temperature. Rhodamine-stained D-MENDs were fabricated by mixing 250 µL of the rhodamine-stained SUV solution and 125 µL of the f-NA core solution followed by forming the vortex and incubating it for 15 min at room temperature. D-MENDs with an additional functionality (R8-MEND) were fabricated by adding STR-R8 in HEPES buffer solution (2 mg ml^−1^) (<20 mol % of the total lipid content) into the bulk batch-fabricated D-MEND solution followed by forming the vortex and incubating it for 15 min at room temperature.

### On-chip Fabrication of MENDs

D-MENDs were fabricated in the microfluidic chip by introducing the SUV solution and the f-NA core solution into separate inlets using microsyringe pumps. Flow rates of the two precursor solutions employed were 3, 5, 7, and 10 µL min^−1^. In each fabrication, the same flow rate was employed for the two precursor solutions. R8-MENDs were fabricated in the microfluidic chip by introducing the bulk batch-fabricated D-MEND solution and the STR-R8 in HEPES buffer solution (2 mg mL^−1^) into respective inlets using microsyringe pumps. Flow rates of 3, 5, 7, and 10 µL min^−1^ were employed for the fabrication. In each fabrication, the same flow rate was employed for the two precursor solutions.

### Characterization

SEM images were obtained with a scanning electron microscope (S-4300, Hitachi, Japan) to observe the structure of the microfluidic chips. The surface of the chip was sputtered with gold for SEM observations. DLS measurements were done with a dynamic light scattering photometer (ELS-Z, Otsuka Electronics, Japan) to estimate size distribution and zeta potential of sample particles. Confocal microscopy measurements were done with a confocal laser scanning biological microscope system (Fluoview FV1000, Olympus, Japan).

## Supporting Information

Figure S1 **DLS particle size distribution profiles for D-MENDs fabricated at various precursor flow rates in the microfluidic chip with a chaotic mixer array.** The profile for D-MENDs fabricated by the bulk SUV fusion method is also included.
(DOCX)

Figure S2 **DLS particle size distribution profiles for R8-MENDs fabricated at various precursor flow rates in the microfluidic chip with a chaotic mixer array.** The profile for R8-MENDs fabricated by the bulk SUV fusion method is also included.
(DOCX)
