# Supplementary material for: Fabrication of Functionalized Double-Lamellar Multifunctional Envelope-Type Nanodevices Using a Microfluidic Chip with a Chaotic Mixer Array
Source: PLoS One. 2012 Jun 18;7(6):e39057. doi: 10.1371/journal.pone.0039057 (PMC3377610; doi:10.1371/journal.pone.0039057)
Supplement: Figure S2 — DLS particle size distribution profiles for R8-MENDs fabricated at various precursor flow rates in the microfluidic chip with a chaotic mixer array. The profile for R8-MENDs fabricated by the bulk SUV fusion method is also included. (DOCX) [file pone.0039057.s002.docx]

**Supporting Information (SI)**

**Fabrication of Functionalized Double-Lamellar Multifunctional Envelope-Type Nanodevices Using a Microfluidic Chip with a Chaotic Mixer Array**

Katsuma Kitazoe, Yeon-Su Park*, Noritada Kaji, Yukihiro Okamoto, Manabu Tokeshi, Kentaro Kogure, Hideyoshi Harashima, Yoshinobu Baba


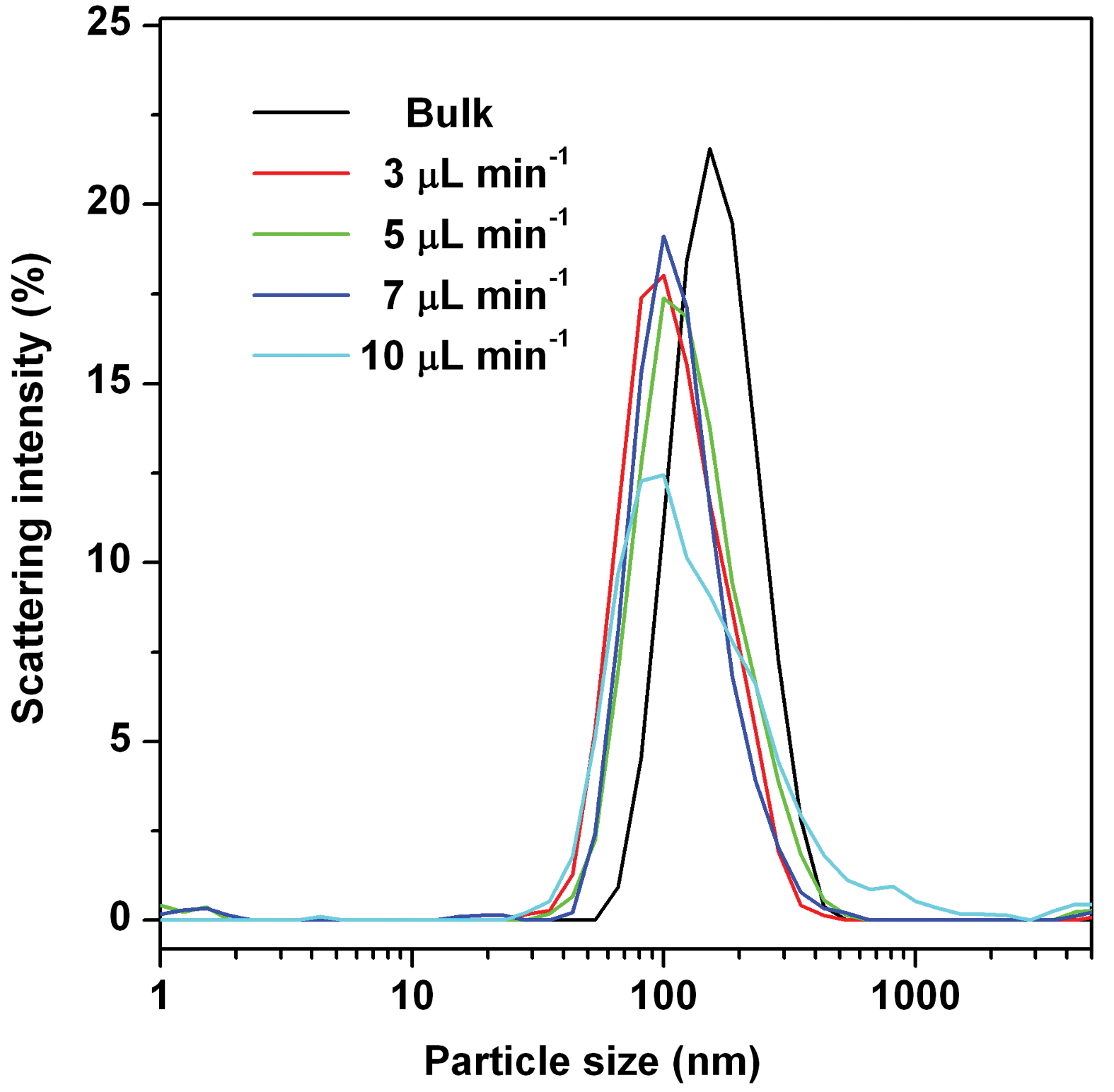


Figure S2. DLS particle size distribution profiles for R8-MENDs fabricated at various precursor flow rates in the microfluidic chip with a chaotic mixer array. The profile for R8-MENDs fabricated by the bulk SUV fusion method is also included.
